# Furniture and television tip-over injuries to children treated in United States emergency departments

**DOI:** 10.1186/s40621-021-00346-6

**Published:** 2021-08-27

**Authors:** Chang Lu, Jaahnavi Badeti, Tracy J. Mehan, Motao Zhu, Gary A. Smith

**Affiliations:** 1 Center for Injury Research and Policy, The Abigail Wexner Research Institute at Nationwide Children’s Hospital, 700 Children’s Drive, Columbus, OH 43205 USA; 2 Northeast Ohio Medical University, Rootstown, OH USA; 3 Department of Pediatrics, The Ohio State University College of Medicine, Columbus, OH USA; 4 Child Injury Prevention Alliance, Columbus, OH USA

**Keywords:** Tip-over, Furniture, Clothing storage unit, Television, Injury, Trauma, Pediatric

## Abstract

**Background:**

Furniture and television tip-over injuries are an important source of injury to children, especially those younger than 6 years old. A current epidemiologic evaluation of tip-over injuries is needed, especially considering changes in the voluntary safety standard for clothing storage units (CSUs) and the shift in the consumer market from cathode ray tube to flat-screen televisions (TVs), and a decline in household TV ownership during recent years. The objective of this study is to update our understanding of the epidemiologic characteristics and trends of furniture (especially CSU) and TV tip-over injuries treated in United States emergency departments among children < 18 years old.

**Methods:**

This study retrospectively analyzed data from the National Electronic Injury Surveillance System from 1990 to 2019. Trends in population-based rates were evaluated with regression techniques. All numbers of cases are expressed as national estimates.

**Results:**

An estimated 560,203 children < 18 years old were treated in United States emergency departments for furniture or TV tip-over injuries during the 30-year study period, averaging 18,673 children annually. CSUs were involved in 17.2% (*n* = 96,321) of tip-overs, and TVs accounted for 41.1% (*n* = 230,325), which included 16,904 tip-overs (3.0%) that involved both a CSU and TV. The rate of furniture and TV tip-over injuries among children < 18 years old increased by 53.8% (*p* < 0.0001) from 1990 to 2010, and then decreased by 56.8% (p < 0.0001) from 2010 to 2019. Almost half (47.0%) of injuries occurred to the head/neck; 3.4% of children were admitted to the hospital. Children < 6 years old accounted for 69.9% of furniture and TV tip-over injuries overall; they accounted for 82.5% of CSU-related and 74.7% of TV-related tip-over injuries.

**Conclusions:**

Despite the decline in tip-over injuries since 2010, more should be done to prevent these injuries, especially among children < 6 years old, because the number of injuries remains high, outcomes can be life-threatening, and effective prevention strategies are known. Safety education, warning labels, and promotion and use of tip restraint devices, while important, are not a substitute for strengthening and enforcing the stability requirements for CSUs and TVs.

## Background

According to the United States (US) Consumer Product Safety Commission (CPSC), there were an estimated 25,500 individuals treated annually, on average, in US hospital emergency departments (EDs) for an injury associated with a tip-over of furniture, televisions (TVs), or appliances from 2017 to 2019 (Suchy, 2021). In addition, there were 571 tip-over-related fatalities reported from 2000 to 2019 (Suchy, 2021). Children are at increased risk of tip-over injury, with most injuries and fatal incidents involving children < 6 years old (Suchy, 2021; Anonymous, 2018; Murray et al., 2009). The number of furniture tip-over injuries among children and adolescents also increased by > 40% from 1990 to 2007 (Gottesman et al., 2009). Clothing storage units (CSUs) are a common source of tip-over injuries, accounting for approximately 2800 injuries among children < 6 years old in 2016 (Anonymous, 2018). TVs are also frequently involved in tip-over injuries, with an average of > 6200 children < 5 years old treated annually in US EDs with these injuries from 1990 to 2011 (De Roo et al., 2013). Traumatic brain injury has been commonly reported among nonfatal and fatal pediatric tip-overs (Suchy, 2021; Murray et al., 2009; Gottesman et al., 2009; De Roo et al., 2013; Yahya et al., 2005; Rutkoski et al., 2011; Lichenstein et al., 2015; Sikron et al., 2006; Bernard et al., 1998).

A current epidemiologic evaluation of tip-over injury trends is needed, especially in light of changes in the ASTM International voluntary safety standard for CSUs, ASTM F2057 (ASTM International, 2019), and the shift in the consumer market from cathode ray tube (CRT) to flat-screen TVs, and a decline in household TV ownership in more recent years (Berry & Woodward, 2017). The objective of this study is to update our understanding of the epidemiologic characteristics and trends of furniture (especially CSU) and TV tip-over injuries among children < 18 years old treated in US EDs, with an emphasis on children < 6 years old. The findings of this study will help inform consumer safety education, product safety standards, and legislative policy to promote prevention of these injuries.

## Methods

### Data source and case selection criteria

Data for patients < 18 years old, who were treated in US EDs for furniture or TV tip-over injuries from 1990 through 2019, were obtained from the National Electronic Injury Surveillance System (NEISS). A case was included in our study if it involved an injury associated with furniture or a TV that fell or tipped onto the patient. NEISS product codes for TVs and furniture included in this study were: 572, 519, 604, 693, 709, 1260, 1684, 1726, 4013, 4014, 4056, 4057, 4065, and 4067. Injuries associated with parts of furniture or non-TV-objects on top of furniture, such as drawers and mirrors, were excluded from the study. The NEISS, which is operated by the CPSC, receives reports from approximately 100 hospitals, which represent a probability sample of the more than 5300 hospitals with a 24-h ED and at least 6 beds in the US and its territories (Schroeder & Ault, 2001). National estimates of ED visits for treatment of injuries associated with consumer products and sports and recreational activities can be calculated from cases reported via the NEISS by applying weights provided by the CPSC, including weights to account for the change in sample design in 1997 (Schroeder & Ault, 2001; Schroeder, 2021; Marker et al., 1999). ED medical records in participating hospitals are reviewed and data are extracted by trained NEISS coders. Data include patient age and sex, products associated with the injury, body region injured, diagnosis, and disposition from the ED. A brief narrative describing the circumstances of the injury is also included for each case.

### Study variables

Using all the recorded product codes for each case, the types of products involved in each case were categorized as (1) furniture and/or (2) TV. Furniture was subcategorized as CSU or non-CSU, based on the ASTM F2057–19 voluntary safety standard, which defines a CSU as a “furniture item with drawers and/or hinged doors intended for the storage of clothing typical with bedroom furniture.” (ASTM International, 2019) CSUs include: dresser, bureau, trunk, chest of drawers, portable closet, armoire, wardrobe, and clothing rack (ASTM International, 2019). Non-CSUs include: table, desk, night stand, entertainment center, cabinet, display/china case, cupboard, hutch, bookshelf/shelfing unit, buffet, shoe rack, TV stand/cart, microwave stand/cart, locker, vanity, grandfather clock, plant stand, pedestal, room divider, safe, and furniture NOS. In this study, “TV-associated” incidents include all events that involved a TV, regardless of whether furniture tipped-over concomitantly with the TV. Incidents involving the concomitant tip-over of a TV and furniture were subcategorized as TV plus CSU or TV plus non-CSU events.

Based on information contained in case narratives, mechanism of injury was categorized as: (1) fell/tipped over, (2) pulled onto self (including “reaching up onto furniture”), (3) climbing furniture (including “standing on top of furniture” and “stepping on furniture”), (4) collision or striking of furniture/knocking over, (5) lifting/moving furniture (including “tried to catch furniture”), (6) opening/closing drawers, (7) pushing on furniture, and (8) found under furniture. Patient age was grouped as: (1) < 6 years, (2) 6–12 years, and (3) 13–17 years. Based on NEISS codes, body region was categorized as: (1) head/neck, (2) upper extremity, (3) lower extremity, and (4) other (including trunk). Diagnosis was grouped as: (1) contusion/abrasion, (2) laceration (including non-dental avulsion), (3) fracture, (4) concussion/closed head injury (CHI), and (5) other. When an injury case listed more than one diagnosis or body region, study analyses included the first-coded diagnosis and body region, which were associated with the most severe injury for that case according to NEISS coding guidelines.” (NEISS, 2021) Location of injury was categorized as: (1) home and (2) other (including school and other public property). Disposition from the ED was grouped into: (1) treated and released, (2) admitted (including treated and transferred to another hospital, treated and admitted within the same facility, and held for < 24-h observation), (3) left against medical advice, and (4) death.

### Data analysis and ethical statement

Data were analyzed using SAS 9.4 software (SAS Institute, Inc., Cary, NC). National estimates were calculated with 95% confidence intervals (CIs). There were 19,045 actual (unweighted) individuals < 18 years old included in this study (13,555 were < 6 years old, 4214 were 6–12 years old, and 1276 were 13–17 years old). All numbers of cases reported throughout the rest of this article are national estimates and all estimates are stable unless stated otherwise. The CPSC considers an estimate to be unstable if the estimate is < 1200, sample size is < 20 actual cases, or coefficient of variation is > 33%. Annual injury rates were calculated using annual population estimates from the US Census Bureau. Simple or piecewise linear regression was performed, as appropriate, to determine whether the slope of the regression line was statistically significantly different from zero to evaluate the secular trends in injury rates during the study period (US Census Bureau, n.d.; US Census Bureau, 2010; US Census Bureau, 2017). The estimated slope (m) from the regression model was reported along with the associated *p*-value. Statistical evaluation included computation of injury proportion ratios (IPRs) with corresponding 95% CIs and *p*-values.

An example of an IPR calculation follows:

$$ \mathsf{IPR}=\left[\left(\mathsf{Number}\ \mathsf{of}\ \mathsf{patients}<\mathsf{6}\;\mathsf{years}\ \mathsf{old}\ \mathsf{with}\ \mathsf{a}\ \mathsf{head}/\mathsf{neck}\ \mathsf{injury}/\mathsf{Total}\ \mathsf{number}\ \mathsf{of}\ \mathsf{patients}<\mathsf{6}\;\mathsf{years}\ \mathsf{old}\right)\times \kern0.37em \mathsf{100}\right]\div \left[\left(\mathsf{Number}\ \mathsf{of}\ \mathsf{patients}\ \mathsf{6}-\mathsf{17}\;\mathsf{years}\ \mathsf{old}\ \mathsf{with}\ \mathsf{a}\ \mathsf{head}/\mathsf{neck}\ \mathsf{injury}/\mathsf{Total}\ \mathsf{number}\ \mathsf{of}\ \mathsf{patients}\ \mathsf{6}-\mathsf{17}\;\mathsf{years}\ \mathsf{old}\right)\times \kern0.37em \mathsf{100}\right] $$

Statistical significance was determined at the α = .05 level. This study was judged to be exempt as non-human research by the Institutional Review Board of the authors’ institution.

## Results

### All tip-overs

#### General characteristics and trends

**Fig. 1:**
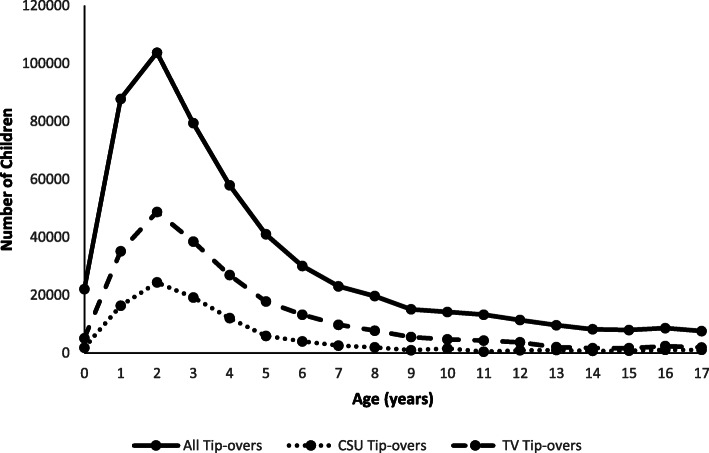
Number of Children Younger Than 18 Years of Age Treated for Tip-over Injuries in United States Emergency Departments by Type of Furniture or Television and by Age, NEISS 1990–2019

**Fig. 2:**
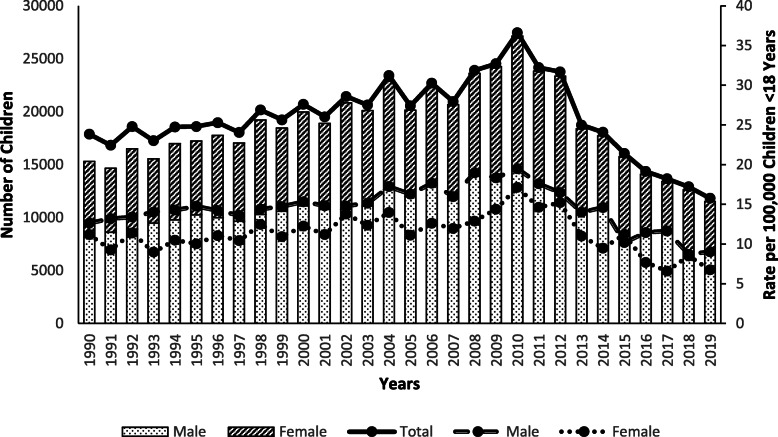
Number and Rate of Children Younger Than 18 Years of Age Treated for Tip-over Injuries in United States Emergency Departments by Sex and by Year, NEISS 1990–2019

An estimated 560,203 children < 18 years old were treated in US EDs for furniture or TV tip-over injuries from 1990 through 2019, averaging 18,673 (range: 11,521–27,140) annually. Boys accounted for 56.2% of cases (Table 1). The location of injury was documented in 78.5% of cases, and of those, 89.9% occurred at home. Children < 6 years old represented 69.9% of injured patients, followed by 6–12-year-olds (22.6%) and 13–17-year-olds (7.5%), with injuries peaking at age 2 years (Fig. 1). The rate of furniture and TV tip-over injuries per 100,000 US population < 18 years old increased by 53.8% (m = 0.5; *p* < 0.0001) from 1990 (23.8) to 2010 (36.6), and then decreased by 56.8% (m = − 2.0; *p* < 0.0001) from 2010 to 2019 (15.8). Although tip-over injuries have decreased since 2010, there were 11,521 children treated in EDs for these injuries in 2019, equaling an average of one child every 46 min. Tip-over injury rates were greater for boys than girls throughout the 30-year study period and both sexes showed similar trends. The rate of tip-over injuries among boys and girls increased by 54.8% (m = 0.3; *p* < 0.0001) and 52.7% (m = 0.2; p < 0.0001), respectively, from 1990 to 2010, followed by a decrease of 53.8% (m = − 1.1; *p* < 0.0001) and 60.2% (m = − 0.9; p < 0.0001), respectively, from 2010 to 2019 (Fig. 2).

**Table 1:** Characteristics of Furniture and Television Tip-over Injuries According to Age Group, NEISS 1990–2019

	Age Groups				
**Characteristics**	< 6 years	6–12 years	13–17 years	< 18 years	
	Number (%)^a^	Number (%)^a^	Number (%)^a^	Number (%)^a^	95% CI
**Mechanism of Injury**					
Fell/tipped over	250,708 (64.0)	106,909 (84.5)	38,081 (91.1)	395,698 (70.6)	323,160 - 468,237
Pulled onto self^b^	76,567 (19.5)	7576 (6.0)	842^*^ (2.0)	84,985 (15.2)	67,603 - 102,366
Climbing furniture^c^	44,351 (11.3)	5430 (4.3)	305^*^ (0.7)	50,086 (8.9)	39,216–60,956
Collision or striking furniture/knocked over	12,717 (3.2)	4049 (3.2)	830^*^ (2.0)	17,596 (3.1)	12,961 - 22,230
Lifting/moving furniture^d^	157^*^ (0.0)	591^*^ (0.5)	1319 (3.2)	2068 (0.4)	1297 - 2840
Opening/closing drawers	671^*^ (0.2)	442^*^ (0.3)	16^*^ (0.0)	1129^*^ (0.2)	592–1666
Pushing on furniture	2691 (0.7)	1381 (1.1)	401^*^ (0.9)	4473 (0.8)	3212 - 5734
Found under	3975 (1.0)	187^*^ (0.1)	6^*^ (0.0)	4168 (0.7)	2532 - 5804
**Body Region Injured**					
Head/neck	209,659 (53.8)	45,013 (35.6)	7556 (18.1)	262,228 (47.0)	216,701 - 307,754
Upper extremity	47,001 (12.0)	27,109 (21.4)	12,128 (29.1)	86,237 (15.5)	69,801 - 102,673
Lower extremity	109,889 (28.2)	46,913 (37.1)	20,130 (48.2)	176,932 (31.7)	142,200 - 211,663
Other	23,271 (6.0)	7497 (5.9)	1923 (4.6)	32,690 (5.8)	26,439 - 38,942
**Diagnosis**					
Contusion/abrasion	163,480 (41.8)	56,437 (44.6)	21,412 (51.2)	241,329 (43.1)	198,872 - 283,786
Laceration	84,719 (21.6)	27,047 (21.4)	6825 (16.3)	118,590 (21.2)	100,102 - 137,078
Fracture	48,110 (12.3)	16,154 (12.8)	5407 (12.9)	69,670 (12.4)	55,610 - 83,731
Concussion/CHI	66,797 (17.1)	13,428 (10.6)	2470 (5.9)	82,695 (14.8)	61,202 - 104,188
Other	28,242 (7.2)	13,446 (10.6)	5679 (13.6)	47,367 (8.5)	33,582 - 61,153
**Type of Furniture or TV**					
TV-associated^e^	172,046 (43.9)	48,751 (38.5)	9528 (22.8)	230,325 (41.1)	187,392 - 273,258
TV alone	151,855 (38.8)	45,573 (36.0)	9452 (22.6)	206,881 (36.9)	168,215 - 245,546
TV + Furniture	20,191 (5.2)	3178 (2.5)	76^*^ (0.2)	23,444 (4.2)	17,987 - 28,901
TV + CSU	14,650 (3.7)	2203 (1.7)	51^*^ (0.1)	16,904 (3.0)	13,289 - 20,518
TV + Non-CSU	5541 (1.4)	975^*^ (0.8)	25^*^ (0.06)	6541 (1.2)	4734 - 8348
CSU^f^	79,511 (20.3)	12,274 (9.7)	4535 (10.8)	96,321 (17.2)	77,577 - 115,064
CSU alone	64,861 (16.6)	10,071 (7.9)	4484 (10.7)	79,417 (14.2)	63,539 - 95,295
TV + CSU	14,650 (3.7)	2203 (1.7)	51^*^ (0.1)	16,904 (3.0)	13,289 - 20,518
Non-CSU^g^	160,471 (41.0)	68,717 (54.3)	27,813 (66.5)	257,001 (45.9)	211,375 - 302,627
Non-CSU alone	154,930 (39.5)	67,742 (53.5)	27,788 (66.5)	250,460 (44.7)	206,005 - 294,915
TV + Non-CSU	5541 (1.4)	975^*^ (0.8)	25^*^ (0.06)	6541 (1.2)	4734 - 8348
**Disposition from Emergency Department**					
Treated and released	371,669 (95.0)	122,768 (97.0)	41,265 (98.7)	535,702 (95.7)	439,516 - 631,889
Admitted	15,467 (4.0)	3078 (2.4)	237^*^ (0.6)	18,781 (3.4)	13,845 - 23,717
Left against medical advice	3526 (0.9)	659^*^ (0.5)	299^*^ (0.7)	4484 (0.8)	2867 - 6101
Fatality	568^*^ (0.1)	7^*^ (0.0)	0^*^ (0.0)	575^*^ (0.1)	202–947
**Sex**					
Male	228,720 (58.4)	67,728 (53.5)	18,155 (43.4)	314,603 (56.2)	257,303 - 371,902
Female	163,092 (41.6)	58,837 (46.5)	23,645 (56.6)	245,574 (43.8)	201,507 - 289,642
**Total**	391,838	126,565	41,800	560,203	

^a^All numbers in this table represent national estimates and all column percentages represent the percentage of total tip-over injuries but do not sum to 100.0% because of rounding error and because of double-counting of injuries involving both TVs and furniture

^b^Includes “reaching up onto furniture;” ^c^Includes” stepping on furniture,” and “standing on top of furniture;” ^d^Includes “tried to catch”

^e^Includes TV tip-overs involving furniture; ^f^Includes TV-associated CSU tip-overs; ^g^Includes TV-associated non-CSU tip-overs

* Estimate is potentially unstable because sample size < 20, estimate is < 1200, or coefficient of variation is > 33%

CHI = closed head injury, CI = confidence interval, CSU = clothing storage unit, TV = television

**Fig. 3:**
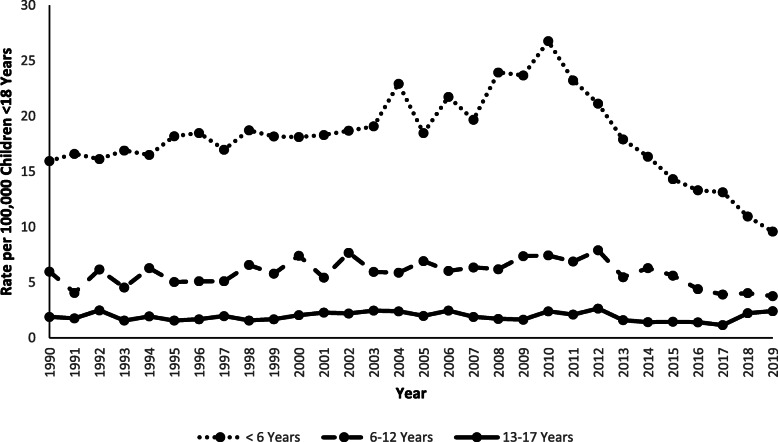
Rate of Children Younger Than 18 Years of Age Treated for Tip-over Injuries in United States Emergency Departments by Age Group and by Year, NEISS 1990–2019

The overall rate of furniture and TV tip-over injuries per 100,000 US population was highest among the < 6-year-old age group, with the rate increasing by 68.6% (m = 0.4; *p* < 0.0001) from 1990 (15.9) to 2010 (26.8), followed by a 64.2% decrease (m = − 1.6; *p* < 0.0001) from 2010 to 2019 (9.6). Among children 6–12 years old, the rate increased by 32.3% (m = 0.09; *p* = 0.0004) from 1990 to 2012, followed by a 52.3% decrease (m = − 0.5; *p* < 0.0001) from 2012 to 2019, while the rate among 13–17-year-olds increased non-significantly by 26.3% (m = − 0.003; *p* = 0.7305) from 1990 (1.9) to 2019 (2.4) (Fig. 3).

**Fig. 4:**
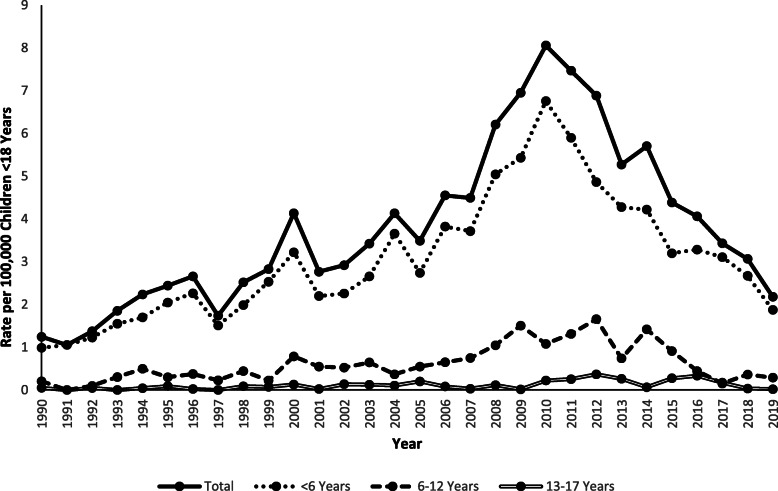
Rate of Children Younger Than 18 Years of Age Treated in United States Emergency Departments for a Concussion/Closed Head Injury Associated with a Tip-over by Age Group and by Year, NEISS 1990–2019

The rate of concussion/CHI associated with furniture and TV tip-overs per 100,000 US population demonstrated a similar trend pattern with an increase of 575.0% (m = 0.3; *p* < 0.0001) from 1990 (1.2) to 2010 (8.1), followed by a decrease of 72.8% (m = − 0.4; p < 0.0001) from 2010 to 2019 (2.2). This trend was driven by children < 6 years old, whose rate of concussion/CHI increased by 580.0% (m = 0.2; p < 0.0001) from 1990 (1.0) to 2010 (6.8), followed by a 72.1% decrease (m = − 0.3; *p* < 0.0001) from 2010 to 2019 (1.9). The rate of concussion/CHI among children 6–12 years old increased by 750.0% (m = 0.06; p < 0.0001) from 1990 (0.2) to 2012 (1.7), followed by a 82.4% decrease (m = − 0.15; p < 0.0001) from 2012 to 2019 (0.3), while the rate among 13–17-year-olds decreased by 60.0% (m = − 0.006; *p* = 0.0026) from 1990 (0.05) to 2019 (0.02) (Fig. 4).

#### Mechanism of injury, body region injured, diagnosis, and disposition

The most common mechanism of injury for all age groups was fell/tipped over (70.6%), followed by pulled onto self (15.2%) and climbing furniture (8.9%) (Table 1). Patients < 6 years old were 3.91 times (IPR 95% CI: 3.82–3.99; *p* < 0.0001) more likely to pull furniture and/or a TV onto themselves than patients 6–17 years old.

Almost half of the injuries occurred to the head or neck region (47.0%), followed by lower extremity (31.7%) and upper extremity (15.5%). Head or neck injuries accounted for most (53.8%) injuries among < 6-year-olds (Table 1), and patients in this age group were 1.72 times (IPR 95% CI: 1.71–1.73; *p* < 0.0001) more likely than patients 6–17 years old to sustain a head or neck injury. Lower and upper extremity injuries were most common among 13–17-year-olds, accounting for 48.2% and 29.1% of injuries in this age group, respectively (Table 1). Overall, contusion/abrasion was the leading diagnosis (43.1%) of patients in this study, followed by laceration (21.2%), concussion/CHI (14.8%), and fracture (12.4%). Most lacerations were to the head/neck (31.9%), and most fractures and contusions/abrasions were to the lower extremity (21.3% and 56.7%, respectively). Patients < 6 years old were 1.81 times (IPR 95% CI: 1.78–1.84; *p* < 0.0001) more likely to sustain a concussion/CHI than patients 6–17 years old.

Overall, 3.4% of children treated in the ED for an injury related to a tip-over required hospital admission, the majority (82.4%) of whom were < 6 years old. Patients < 6 years old were 2.01 times (IPR 95% CI: 1.93–2.08; p < 0.0001) more likely to be admitted than patients 6–17 years old. Patients with a fracture were 6.89 times (IPR 95% CI: 6.70–7.08; p < 0.0001) and patients with a concussion/CHI were 2.38 times (IPR 95% CI: 2.31–2.46; p < 0.0001) more likely to be admitted to the hospital than patients with other diagnoses.

There were an estimated 575 fatalities in this study, of which, 98.8% occurred among children < 6 years old. Similar to nonfatal tip-over injuries, fatalities peaked at age 2 years. More than half of tip-over deaths (51.0%, *n* = 293) were associated with an injury to the head/neck. Half of fatalities (50.1%, *n* = 288) were associated with a CSU tip-over and more than one-third of fatalities (35.0%, *n* = 201) involved a TV tip-over. Because these national fatality estimates are potentially unstable, they should be interpreted with caution and further sub-analyses were not performed.

### Clothing storage unit tip-overs

An estimated 96,321 children < 18 years old were treated in US EDs for CSU tip-over injuries from 1990 through 2019, averaging 3211 (range: 2038–5020) annually. CSUs were involved in 17.2% of all tip-over injuries, which included 16,904 tip-over injuries (3.0%) that involved both a CSU and TV (Table 1). Boys accounted for 55.6% of CSU tip-over cases, and 4.2% of children injured by a CSU tip-over were hospitalized. Children < 6 years old represented 82.5% of patients injured by CSU tip-overs, followed by 6–12-year-olds (12.7%) and 13–17-year-olds (4.7%), with injuries peaking at age 2 years (Fig. 1). Patients < 6 years old were 2.03 times (IPR 95% CI: 2.00–2.06; *p* < 0.0001) more likely to sustain a CSU-related tip-over injury than patients 6–17 years old. Injuries to the head/neck accounted for 58.2% of all CSU tip-over injuries, and among patients < 6 years old, they accounted for 65.0% of CSU tip-over injuries. Concussion/CHI was the diagnosis in 19.7% of CSU tip-over cases, and among children < 6 years old, concussion/CHI was the diagnosis in 22.0% of cases. CSU tip-overs were 1.44 times (IPR 95% CI: 1.41–1.46; p < 0.0001) more likely to result in a concussion/CHI than tip-overs not involving CSUs. In addition, CSU tip-overs were 1.30 times (IPR 95% CI: 1.26–1.35; p < 0.0001) more likely to result in hospital admission than tip-overs not involving CSUs.

**Fig. 5:**
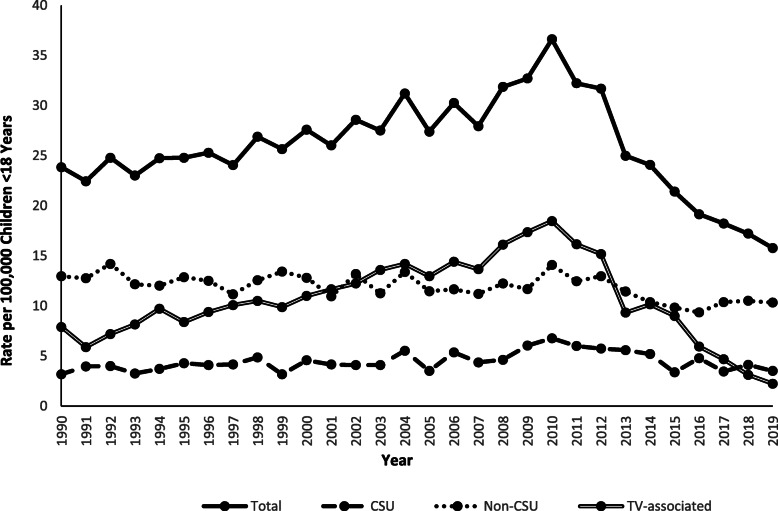
Rate of Children Younger Than 18 Years of Age Treated for Tip-over Injuries in United States Emergency Departments by Type of Furniture or Television and by Year, NEISS 1990–2019

The rate of injuries per 100,000 US population < 18 years old attributable to CSU tip-overs increased by 112.5% (m = 0.11; p < 0.0001) from 1990 (3.2) to 2010 (6.8), followed by a 48.5% decrease (m = − 0.2; *p* = 0.0005) from 2010 to 2019 (3.5). This trend pattern contrasted with that for the rate of injuries related to non-CSU tip-overs, which decreased by 20.2% (m = − 0.08; *p* = 0.0003) from 1990 (12.9) to 2019 (10.3) (Fig. 5). Although CSU tip-over injuries have decreased since 2010, there were 2560 children treated in EDs for these injuries in 2019, averaging one child every 3.4 h.

### Television tip-overs

An estimated 230,325 children < 18 years old were treated in US EDs for TV tip-over injuries from 1990 through 2019, averaging 7677 (range: 1630–13,690) annually. TV was the most common product category involved in tip-over injuries (41.1%), and among those TV-associated injuries, 10.2% (*n* = 23,444) also involved concomitant tip-over of furniture, with CSUs accounting for a majority (*n* = 16,904) of these cases. Boys accounted for 57.3% of TV tip-over cases, and 4.5% of children injured by a TV tip-over were hospitalized. Children < 6 years old accounted for 74.7% of patients injured by TV tip-overs, followed by 6–12-year-olds (21.2%) and 13–17-year-olds (4.1%), with injuries peaking at age 2 years (Fig. 1). Patients < 6 years old were 1.27 times (IPR 95% CI: 1.26–1.28; *p* < 0.0001) more likely to sustain a TV-associated tip-over injury than patients 6–17 years old. Injuries to the head/neck accounted for 50.8% of TV tip-over injuries among all age groups overall and 53.4% among < 6-year-olds. In addition, concussion/CHI was diagnosed in 19.4% of TV tip-over cases overall, and in 20.6% of the subgroup of children < 6 years old. TV-associated tip-overs were 1.69 times (IPR 95% CI: 1.67–1.71; *p* < 0.0001) more likely to result in a concussion/CHI than tip-overs not associated with a TV. Additionally, patients with TV tip-over injuries were 1.75 times (IPR 95% CI: 1.71–1.80; p < 0.0001) more likely to be admitted to the hospital than patients with a tip-over injury not associated with a TV.

**Fig. 6:**
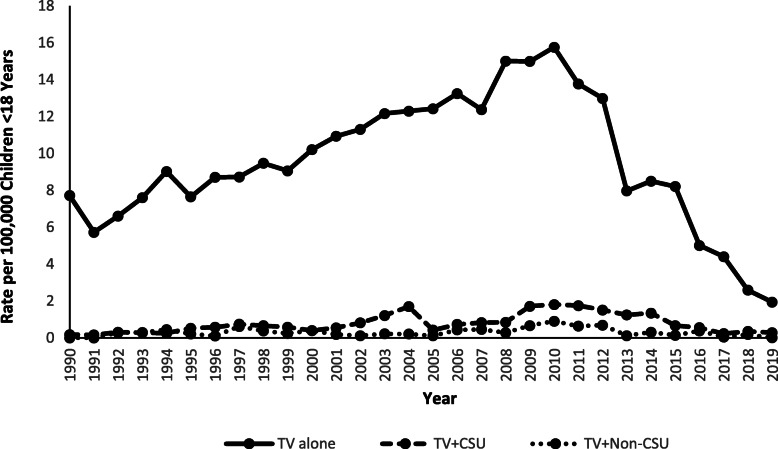
Rate of Children Younger Than 18 Years of Age Treated for Television Tip-over Injuries in United States Emergency Departments by Association with Concomitant Tip-over of Clothing Storage Units or Non-Clothing Storage Units and by Year, NEISS 1990–2019

The rate of TV-associated tip-over injuries per 100,000 US population < 18 years old increased by 134.2% (m = 0.5; p < 0.0001) from 1990 (7.9) to 2010 (18.5), followed by a 88.1% decrease (m = − 1.7; p < 0.0001) from 2010 to 2019 (2.2) (Fig. 5). Likewise, the rate of injuries associated with “TV alone” tip-overs increased by 105.2% (m = 0.4; p < 0.0001) from 1990 (7.7) to 2010 (15.8), followed by a 88.0% decrease (m = − 1.5; p < 0.0001) from 2010 to 2019 (1.9) (Fig. 6). The rate of injuries related to concomitant tip-overs of a TV plus a CSU was variable during 1990 to 2010, increasing overall by 800.0% (m = 0.07; p < 0.0001) from 1990 (0.2) to 2010 (1.8), followed by a decrease of 83.3% (m = − 0.13; p < 0.0001) from 2010 to 2019 (0.3) (Fig. 6). There were no reported injuries associated with concomitant tip-overs of a TV plus a non-CSU in 1990 and 1991; however, the rate increased by 200.0% (m = 0.02; *p* = 0.003) from 1992 (0.3) to 2010 (0.9), followed by a 99.3% decrease (m = − 0.05; *p* = 0.002) from 2010 to 2019 (0.006) (Fig. 6). Although TV-associated injuries have decreased since 2010, there were 1630 children treated in EDs for these injuries in 2019, averaging one child every 5.4 h.

## Discussion

### All tip-overs

An estimated 560,203 children < 18 years old were treated in US EDs for furniture or TV tip-over injuries from 1990 through 2019. Although the rate of these injuries has decreased since 2010, the number remains high. The observed trends were driven by children < 6 years old, who represented approximately 70% of injured patients and experienced the highest injury rates. Children < 6 years old were also disproportionately represented among patients admitted to the hospital, accounting for 82% of admissions. These young patients were twice as likely to be admitted than those 6–17 years old. In addition, although the estimate was potentially unstable, children < 6 years old represented about 99% of deaths. These findings are consistent with those from previous reports (Suchy, 2021; Murray et al., 2009; Gottesman et al., 2009; De Roo et al., 2013).

The observed injury patterns are attributable to the behavioral and physical characteristics of young children. Young children spend much of their time in the home around furniture and TVs. They are curious and often do not recognize potential danger. For example, they pull themselves up on furniture to reach an attractive object placed on top or use drawers as steps to climb CSUs (Therrell et al., 2002). They also have inadequate physical and cognitive abilities to make rapid injury avoidance responses once a tip-over is initiated or to extricate themselves if trapped under a fallen object. Because of their short stature, they are more likely to sustain head/neck injuries than older children, who more commonly experience an injury to the lower extremities. Head/neck injuries accounted for 54% of injuries among < 6-year-olds, whereas lower extremity injuries represented 48% of injuries among 6–17-year-olds in this study. In addition, patients < 6 years old were 1.81 times more likely to sustain a concussion/CHI than patients 6–17 years old. The overall rate of concussion/CHI, which is among the most serious injury diagnoses in this study based on admission rates, has decreased since 2010, but remains high in the younger age group. These findings are consistent with those reported previously (Suchy, 2021; Murray et al., 2009; Gottesman et al., 2009; De Roo et al., 2013).

### Clothing storage unit tip-overs

CSU tip-over injuries represent an important preventable subset (17.2%) of all tip-over injuries. An estimated 96,321 children < 18 years old were treated in US EDs for CSU tip-over injuries during the 30-year study period. Children < 6 years old represented 82.5% of patients injured by CSU tip-overs. Two voluntary safety standards, ASTM F2057–19 and ASTM F3096–14, specify performance requirements to help prevent these tip-over events (ASTM International, 2019; ASTM International, 2014). ASTM F2057–19 is intended to protect children up to and including 5 years of age and requires that CSUs pass two stability tests; specifically, they must not tip over 1) when all drawers or doors are opened while the unit is empty, and 2) when one drawer or door is opened at a time and a 50 pound weight is hung on the front of that drawer or door (ASTM International, 2019). Many organizations, including the American Academy of Pediatrics, Kids in Danger, Parents Against Tip-overs, Consumer Federation of America, and Consumers Union, have raised concerns that a 50-pound test is not sufficient (American Academy of Pediatrics, 2018; Kids in Danger, 2016; US Consumer Product Safety Commission, 2017a; Comments of Consumer Reports to the Consumer Product Safety Commission on the Advance Notice of Proposed Rulemaking, 2018). Sixty pounds represents the 95th percentile weight of a 72-month-old child and therefore more adequately covers the weight range of the age group at risk of injury from tip-overs (Centers for Disease Control and Prevention, 2002). Staff at the CPSC also support an increase of the test weight to 60 pounds (US Consumer Product Safety Commission, 2016). However, a proposal to increase the test weight to 60 pounds was rejected by members of the ASTM Subcommittee F15.42 on Furniture Safety, a decision supported by the American Home Furnishings Alliance, representing more than 200 furniture manufacturers and distributors worldwide (Ledoux, n.d.). While furniture manufacturers have voiced concerns about the potential costs of implementing a more rigorous stability test (Peachman, 2018), many designs already on the market are stable enough to meet the 60-pound requirement and at least one retails for less than $100 (Anonymous, 2018). In fact, one study demonstrated that many tested units remained stable when > 70 pounds were added to an open drawer (Kids in Danger, 2016).

ASTM F2057–19 specifies that stability testing be done on a “hard, level, flat surface.” (ASTM International, 2019) However, this is not representative of the types of surfaces on which CSUs are placed in many homes. In fact, the CPSC found that among nonfatal CSU tip-over incidents that specified the type of flooring involved, most occurred on carpet (US Consumer Product Safety Commission, 2017a). In addition, the current safety standard only applies to CSUs that are 27 in. or more in height, which leaves shorter ones without minimum tip-over performance requirements. The safety standard also does not address the common scenarios of drawers being filled with typical items or multiple drawers simultaneously being opened and a downward force being applied. CSU stability should be tested with the drawers filled with typical items, and a progressive drawer-opening stability test also should be required of CSUs; units that fail to pass should be required to include drawer interlock systems (Kids in Danger, 2016).

ASTM F2057–19 also specifies that a tip restraint device must be provided with each CSU at the point of sale; this is usually a strap that the consumer must attach between the CSU and a wall. Another safety standard, ASTM F3096–14, specifies the test method and other requirements for these tip-over restraints, including that they can withstand a pull force of 50 pounds (ASTM International, 2014). An anchoring system will prevent tip-overs, but only if it is installed correctly. Unfortunately, many consumers choose to not anchor their furniture to the wall for various reasons. Families living in rental homes may not be allowed to drill holes in the wall. Some may lack the necessary tools or skills for affixing the anchors, while others are simply unaware of the tip-over hazard and the potential severity of the consequences. One possible solution is adhesive-backed tip restraint devices that have one end of the restraint system attached to the furniture before it leaves the factory (Butturini et al., 2015). This would reduce the skills required of consumers, avoid drilling holes into walls, and raise immediate attention to the hazard.

The CPSC launched the “Anchor It!” educational campaign in 2015 to improve awareness of the tip-over hazard (US Consumer Product Safety Commission, 2015). Other child safety groups also have engaged in educational outreach to consumers. Warning labels are another strategy for raising awareness of a hazard, and ASTM F2057 specifies requirements of warning labels on CSUs (ASTM International, 2019). However, the CPSC reported in 2016 that only 56% of 61 CSUs it evaluated contained a warning label related to tip-over hazards and only 8% of the CSUs had labels that were fully compliant with the voluntary standard (US Consumer Product Safety Commission, 2016). Although warning labels are a common strategy favored by manufacturers, warnings are not adequately effective in influencing consumers’ perceptions of risks or changing their behaviors (Argo & Main, 2004). Tip restraint devices, consumer educational programs, and warning labels are part of a multi-pronged prevention approach, but they are not a substitute for improving and enforcing furniture stability.

Despite widely publicized legal settlements and recalls by the CPSC (Domonoske, 2016; Bromwich, 2016),_ENREF_29 _ENREF_31compliance of CSUs on the market with the voluntary safety standard is problematic. A study released in 2016 by the CPSC revealed that of 61 CSUs sampled, 51% did not comply with stability requirements and 30% did not include a tip restraint device with their product as required by the safety standard. The identified tip-over restraints that were provided were all strap-style devices that require anchoring (US Consumer Product Safety Commission, 2016). These findings agree with another 2016 study that found that only 47% of 19 dressers and chests that were tested passed the ASTM F2057 performance tests (Kids in Danger, 2016). Consumers cannot determine the stability of a CSU by looking at it; therefore, manufactures need to comply with adequate safety standards and compliance must be enforced by the CPSC. In response to on-going deaths and injuries of young children associated with CSU tip-overs, the CPSC initiated a rulemaking process in 2017 to address these events. In the advance notice of proposed rulemaking, the CPSC stated that “the Commission preliminarily believes that the ASTM standards do not adequately reduce the risk of injury associated with CSU tip-overs.” (US Consumer Product Safety Commission, 2017a)

In addition to voluntary standards and CPSC regulations, federal legislation can be used to prevent consumer product injuries. HR 2211, known as the “Stop Tip-overs of Unstable, Risky Dressers on Youth Act” or the “STURDY Act,” was passed by the US House of Representatives in September 2019, but was not passed by the US Senate before the end of the 116th Congress (H.R.2211, n.d.). It would have required the CPSC to promulgate a consumer product safety rule to protect children < 6 years old from CSU tip-over injuries and death. This rule would have represented a mandatory standard rather than the voluntary ASTM F2057 standard and would have applied to all CSUs regardless of height, applied a 60 pound weight for stability testing, and expanded testing to better simulate real world conditions, such as carpeted surfaces, drawers containing items, multiple open drawers, and dynamic force (H.R.2211, n.d.). The legislation was reintroduced in February 2021 in the 117th Congress as HR 1314 and S 441. The US House of Representatives passed HR 1314 in June 2021 (H.R. 1314, n.d.).

### Television tip-overs

TV tip-over injuries accounted for 41.1% of all tip-over injuries, with an estimated 230,325 children < 18 years old treated in US EDs for TV tip-over injuries during the 30-year study period. Among these TV-associated injuries, 10.2% (*n* = 23,444) involved concomitant tip-over of furniture, with CSUs accounting for a majority (*n* = 16,904) of these cases. This agrees with previous reporting of tip-over fatalities that demonstrated the common combination of a TV and CSU tipping over together (US Consumer Product Safety Commission, 2017a). Children < 6 years old accounted for three-fourths (74.7%) of patients injured by TV tip-overs. This is consistent with other reports, including from other countries, such as Canada, where 70% of TV tip-overs involve children 1–3 years old (Health Canada, 2017).

The decline in the overall rate of furniture and TV tip-over injuries beginning in 2010 was driven by the decline in the rate of TV-associated injuries, and especially injuries involving a tip-over of a TV alone without other furniture. This trend was particularly prominent among children < 6 years old. This observed decline in TV tip-over injuries may be attributable to several factors. There has been a decrease in the average number of televisions in US homes from 2.6 televisions per household in 2009 to 2.3 televisions in 2015 (Berry & Woodward, 2017), which is believed to be due to an increasing number of smart devices that can stream video content. In addition, a 2017 CPSC report found that 91% of injuries associated with TVs falling off furniture involved CRT TVs (US Consumer Product Safety Commission, 2017b). CRT TVs are typically heavier towards the front than the rear, making them more prone to tip forward. In addition, flat-screen TVs are much lighter than CRT TVs with a similar screen size. CRT TVs are no longer manufactured; therefore, the observed decline in TV tip-over injuries may be, in part, attributable to the gradual disappearance of older CRT TVs from homes as they are replaced by flat-screen TVs.

The UL 60065 safety standard “Audio, Video, and Similar Electronic Apparatus – Safety Requirements” specifies the stability performance requirements for TVs (Underwriters Laboratories, 2015). The stability test conditions vary based on TV parameters, such as weight, height, and screen size, and include a tilt test, vertical force test, and horizontal force test. Unlike ASTM F2057, the UL 60065 standard does not include a requirement that a tip restraint device be sold with a TV. Unanchored TVs in the home are unsafe; therefore, TVs should be sold with appropriate safety anchors, just as required for CSUs. In addition, Underwriters Laboratories has a separate standard, UL 1678, that includes stability requirements for carts, stands, and entertainment centers used to support TVs and similar equipment. The tip stability performance test in UL 1678 places the cart or stand on a 10-degree incline and applies a simulated TV load (Underwriters Laboratories, 2019). Cart and stand stability requirements have also been included in the most recent version of UL 60065 (Underwriters Laboratories, 2015).

Parents should not place TVs on furniture, such as a CSU, that is not designed to support a TV. A TV that is placed on appropriate furniture should be anchored to the wall along with the furniture supporting it. TV remote controls, toys, or other attractive items should not be placed on top of a TV or the furniture supporting it because this may encourage climbing by young children, resulting in a tip-over.

### Study limitations

This study has several limitations. It underestimates the true number of furniture and TV tip-over injuries because only cases treated in EDs were included. Injuries for which medical treatment was not sought and injuries treated in other medical settings, such as urgent care facilities or private physician offices, are not captured by the NEISS. Fatalities are also not captured well because many are not transferred to an ED. NEISS case narratives may have inconsistent or missing documentation of details, which could lead to mis-categorization of injuries. Because of limited documentation, we were unable to determine the height of the furniture, the type of TV (CRT versus flat-screen), whether a tip restraint device was used, or the type of floor on which the furniture was placed. Despite these limitations, the strength of our study lies in its use of a nationally representative sample of tip-over injuries over a 30-year period.

## Conclusions

Despite the decline in tip-over injuries since 2010, more should be done to prevent these injuries, especially among children < 6 years old, because the number of injuries remains high, outcomes can be life-threatening, and effective prevention strategies are known. Safety education, warning labels, and promotion and use of tip restraint devices, while important, are not a substitute for strengthening and enforcing the stability requirements for CSUs and TVs.

## Data Availability

Data analyzed in this study were from the National Electronic Injury Surveillance System, which is managed by the United States Consumer Product Safety Commission (CPSC). Data requests should be submitted to the CPSC.

## Abbreviations

CI: Confidence Interval
CPSC: United States Consumer Product Safety Commission
CRT: Cathode Ray Tube
CSU: Clothing Storage Unit
ED: Emergency Department
IPR: Injury Proportion Ratio
NEISS: National Electronic Injury Surveillance System
TV: Television
US: United States
